# Neuritis: Its Definition and Successful Treatment

**Published:** 1915-06

**Authors:** Naunton Davies

**Affiliations:** Llandrindod Wells


### NEURITIS: ITS DEFINITION AND SUCCESSFUL TREATMENT.

BY

NAUNTON DAVIES, F.R.C.S.,
*Llandrindod Wells.*

FOR practical purposes I should define neuritis as: "Pain in one or more nerves due to changes—morbid conditions—in the nerve or nerves at the seat of pain, and not to general or remote causes." By the *seat of pain* I mean the actual site of mischief, and not any part of a nerve sympathetically affected.

I propose placing in a separate class those cases of nerve disturbance due to general or remote causes, under the term "pseudo-neuritis," or, if it is preferred, "sympathetic neuritis." As examples of pseudo-neuritis I may refer to the lightning pains of locomotor ataxia in the limbs (due to a remote cause), and to the burning, shooting pains often met with in diabetes (due to a general cause, toxæmia). Cancer supplies another instance of this class of neuritis. Curiously enough, the pain of neuritis associated with cancer is aggravated by the treatment which cures true neuritis; and this phenomenon enables one to diagnose cancer long before any visible sign of that disease manifests itself. One of the worst forms of pseudo-neuritis we find associated with Hodgkin's disease.

No single method of treatment can be applied in these cases, which need, of course, individual study and special attention.

Then there are cases due to the direct pressure of bony growths or tumours upon nerves, the treatment for which is too obvious to need remark.

After excluding these cases of pseudo-neuritis, there remain the cases I shall call "true neuritis" to be dealt with. They form a very large percentage of neuritis cases, which are all, fortunately, curable. This statement may appear to verge on the extravagant, but it is based on an experience extending over a period of sixteen years and a long series of cases.

In true neuritis, simple or multiple, the pain is due to hyperæmia, exudations or adhesions—generally to all three—affecting a nerve and its sheath ; and, as a rule, the morbid area or areas are limited in extent, and can be traced to a spot or spots usually no larger than a split pea, and sometimes even smaller. These morbid spots may be widely separated, or may lie comparatively near to each other, but whatever the distances of separation, there is always a space between them free from trouble. Each area is an independent, exciting cause of pain. Consequently it follows that every diseased spot in an affected nerve must be localised and treated directly before neuritis can be got rid of. If one spot of trouble is overlooked the pain remains in that spot, and may radiate over a wider field (sympathetic or reflected pain), even extending to and covering the areas previously treated, and giving rise to a feeling on the part of the patient that no good has been done, although perhaps nine-tenths of the mischief have been got rid of.

In some of the most severe cases agonising pain may arise from one diseased spot, radiating over a network of nerves, and when this spot has been treated the pain permanently disappears, often within an hour or two of treatment.

It seems to me that the term "simple neuritis" should be used to describe neuritis due to one diseased spot, and "multiple neuritis" to describe neuritis due to more than one diseased spot, whether these spots are in one nerve or several.

Uniformly satisfactory treatment can only be assured by strict observance of the *technique,* which I shall now describe.

*Testing for Neuritis.*—Attach a small conical electrode to the negative pole of a Faradic battery, and an ordinary disc electrode (or chain electrode) about two inches in diameter to the positive pole. Soak the electrodes in warm saline solution, and apply the positive to the skin at a spot remote from the seat of pain. The negative is then applied to an indifferent point, and the current turned on until the patient feels it distinctly.

The current should be just strong enough to excite pain in a diseased nerve-spot, but not to cause pain in a healthy nerve. It is most important to observe this point carefully, otherwise the test will be misleading.

The negative electrode is now moved over the skin slowly towards the suspected field of mischief. Directly a "spot" is touched the patient is conscious of an intensified sensation, and may wince more or less, according to the acuteness of the underlying mischief and the strength of the current. It is well to approach this responsive spot from different points, as travellers converge upon cross-roads from various directions, and if the finger-post of pain always indicates the same spot, the conclusion is obvious. In this way the whole nerve-field in the neighbourhood of the mischief is explored, and the worst of the painful spots marked for treatment. A little experience enables one to feel sure of the actual state of things, and to distinguish between the reaction at the seat of mischief and the mild sensation provoked over a sympathetic area. If the patient is of the hysterical type, the testing must be carried out with extra care.

A local anæsthetic is next injected, thoroughly infiltrating the nerve and neighbouring tissues. When prolonged anæsthesia is desirable in specially nervous or worn-out patients, hydro-chloride of urea and quinine is most useful.

Personally I make it a rule to administer by the mouth $\frac{1}{150}$ grain of trinitrine, $\frac{1}{100}$ grain digitaline, and a teaspoonful of aromatic spirit in water before giving the hypodermic, to obviate the unpleasant after-effects of cocaine, and it answers its purpose admirably.

When anæsthesia is complete, the disc or chain electrode already described is attached to the positive pole of a *galvanic battery*, and placed on an indifferent part some inches —not less than three or four—away from the spot to be treated. An insulated needle, with a platinum spear-point, exposed for one-third to one-eighth [1] of an inch, carried in a suitable handle, is attached to the negative pole, and held against the spot marked. The current is turned on very gradually, increased slowly up to, but *never more*, than 6 m.a., and the needle-point is pressed gently through the skin towards the underlying nerve.

Some patients are very sensitive, in spite of the anæsthetic, and cannot bear the full dose (6 m.a.), but in these cases the lesser dose is sufficient to bring about the desired result. The current is to be continued for *ten minutes*, and turned off very slowly, for sudden stoppage causes pain.

[1] Needles for nerves near the surface should have a short "spear," to avoid damaging the skin.

The needle should be pushed through the skin beyond the spear-head as quickly as possible, for the skin sometimes retains a little sensitiveness, and it is well to guard against electrolytic action upon the skin, by bringing it into contact with the insulated portion of the needle. In this way you avoid making unnecessarily large punctures. Once through the skin, the needle is worked towards the affected spot in the nerve, its approach being signalled by slight twitchings of the muscles and increased sensation, the patient often remarking: "That is the exact spot. It is like the old pain." When you succeed in causing the old pain during the treatment, you may be sure that your *technique* is right.

The rule is: Where sensation is most acute there the treatment must be concentrated, directing the current upon it from the point of the needle, allowing the latter to touch or hover about the nerve, but not to pass through it. Here experience along can guide you, aided by close observation of muscular reactions and the patient's signals.

The punctures left by the needles should be painted over with a layer or two of "new skin" or liquid court plaster.

Needles of various lengths are required—from one to five inches—according to the depth of the nerve to be treated beneath the surface.

I have never known any ill-effects following treatment. It must not, however, be forgotten that atrophy of a nerve and more or less paralysis are often the sequelæ of neuritis. They should be noted *before treatment*, and pointed out to the patient, otherwise the mischief may be attributed to the wrong cause.

To explain the beneficial results of this treatment, it is only necessary to point out the effect of electrolytic action upon hyperæmia, exudations and adhesions, and the effect of adjusted doses of current upon excited nerve-centres and inert physiological conditions.

The question has often been put to me : "Why does this treatment succeed in cases where excision has failed ?" The answer is simple. The diseased spot in neuritis is very small, and, in the absence of testing and isolation, is often missed, and a healthy portion of the nerve excised. Testing directs the needle unerringly to the spot, and the good result follows.

It may be interesting to refer to a few cases treated by this method :—

Mrs. D., aged 50, severe neuritis of the fifth nerve, of two years' duration, during the whole of which time she had been under treatment. In London she had ethereal and other injections, and the varied treatment usual in such cases, but without any good result. All her teeth had been extracted. As her general health was rapidly failing, under the stress of pain and insomnia, she was advised to have her Gasserian ganglion excised. In this condition she presented herself to me for treatment.

After cocainising, a needle was passed through the mental foramen into the inferior dental canal, the positive disc applied to the temple, and the procedure already described followed out. After an interval of a day the auriculo-temporal nerve was treated in the same way, and then, another day intervening, the frontal, the facial and a communicating branch of the temporal. The patient was now able to rest at night, and take more food, but felt occasional sharp paroxysms of pain in the lower jaw. A sensitive spot was found on the edge of the gums. This was treated with a cutting cautery, the gums being slit down to the bone for an inch or so. The last remnant of pain disappeared, sleep returned, the general health rapidly improved, and there has been no relapse. It ought to be mentioned that there was a little catarrh of the middle ear in this case, and that some neboline, No. 8, was injected into the chamber through the Eustachian.

This case is important, as it shows that the disease had not invaded the Gasserian ganglion, and that the removal of the ganglion was unnecessary. Indeed, it would have resembled the uprooting of a tree for canker in some of its branches when pruning would have done. I was not hopeful of good results in this case, owing to the difficulty of getting at the nerve in the dental canal and to adverse anatomical conditions. To take a simpler case :—

Neuritis of an intercostal nerve, in a well-built, athletic man of 48, who had been treated in South Africa and afterwards in London, without benefit. Testing revealed one spot of trouble only, which was immediately treated. In less than an hour the pain, which had been intense and constant, had quite gone. In three days the patient was playing golf.

Another case :—

A builder of 35, whose case had been diagnosed in London as "disease of the membranes of the brain," came to Llandrindod Wells, as he thought and was told, to end his days drinking the waters ! It turned out to be neuritis of the extra-cranial nerves. Seven spots were localised, and treated on alternate days. The pain cleared up in three weeks, and the patient has had no relapse since the treatment, four years ago.

One more case :—

That of a man of middle age, suffering intense pain in the head, the paroxysms being so severe that he had to be prevented by force from running his head against the walls of his room. He had had portions of his nerve excised, and two years' treatment in hospitals and out, without obtaining even temporary relief. Seven painful spots were found in his scalp, which were treated on alternate days in the usual way. He got quite well in a few weeks, and returned to his work. That was nine years ago, and there has been no relapse.

Enough has been said to indicate the value of this treatment in varied forms of neuritis.

In neuritis of the extremities the procedure is comparatively simple, and the results are invariably good.

It would be rash to assume that every case of neuritis of the "fifth" can be cured by this or any other method. The anatomical conditions are so obstructive that one can conceive cases in which the "spots" are so placed as to defy access. At the same time, I have not so far met with any of these intractable cases.

To summarise : Every painful spot in a nerve or nerves must be accurately localised, marked, and anæsthetised. The needle must be long enough to reach the affected nerve, and be inserted into the exact spot, otherwise the result will be disappointing. The current must be slowly turned on and off, and never allowed to continue for more than ten minutes, at a maximum dosage of 6 m.a. The needle puncture must be covered with aseptic varnish. Severe cases may be treated daily, subacute or chronic cases on alternate days. The patient should rest for a few hours after treatment.

In acute sciatica rest and treatment should go on hand-in-hand for a few days, until the worst of the pain has been subdued, and then quiet and increasing exercise should be advised, followed by the Scotch douche.

There are, of course, the questions of exciting causes and general treatment to be considered, but these lie outside the scope of this article.

A word of caution as to the risk of ordering prolonged rest in neuritis of a limb, which often leads to contraction of a joint and permanent stiffness. This is a serious matter, and as the pain for which rest is prescribed can be quickly got rid of by other means, there seems to be no justification for the "rest cure." Massage too does much harm in acute neuritis, and should only be resorted to to restore wasted muscles or stiff joints, after the pain has ceased.

NOTE.—Portions of this article appeared in *The Practitioner*, June, 1914 ("Neuritis: Treatment by Electricity)."

